# Transcriptomic Analysis of Trout Gill Ionocytes in Fresh Water and Sea Water Using Laser Capture Microdissection Combined with Microarray Analysis

**DOI:** 10.1371/journal.pone.0139938

**Published:** 2015-10-06

**Authors:** Isabelle Leguen, Aurélie Le Cam, Jérôme Montfort, Sandrine Peron, Alain Fautrel

**Affiliations:** 1 INRA, UR1037 Fish Physiology and Genomics, Rennes, France; 2 INSERM UMR991, Rennes, France; 3 Université de Rennes 1 Plateforme H2P2, Biosit, Rennes, France; Centre of Marine Sciences & University of Algarve, PORTUGAL

## Abstract

Fish gills represent a complex organ composed of several cell types that perform multiple physiological functions. Among these cells, ionocytes are implicated in the maintenance of ion homeostasis. However, because the ionocyte represents only a small percent of whole gill tissue, its specific transcriptome can be overlooked among the numerous cell types included in the gill. The objective of this study is to better understand ionocyte functions by comparing the RNA expression of this cell type in freshwater and seawater acclimated rainbow trout. To realize this objective, ionocytes were captured from gill cryosections using laser capture microdissection after immunohistochemistry. Then, transcriptome analyses were performed on an Agilent trout oligonucleotide microarray. Gene expression analysis identified 108 unique annotated genes differentially expressed between freshwater and seawater ionocytes, with a fold change higher than 3. Most of these genes were up-regulated in freshwater cells. Interestingly, several genes implicated in ion transport, extracellular matrix and structural cellular proteins appeared up-regulated in freshwater ionocytes. Among them, several ion transporters, such as CIC2, SLC26A6, and NBC, were validated by qPCR and/or in situ hybridization. The latter technique allowed us to localize the transcripts of these ion transporters in only ionocytes and more particularly in the freshwater cells. Genes involved in metabolism and also several genes implicated in transcriptional regulation, cell signaling and the cell cycle were also enhanced in freshwater ionocytes. In conclusion, laser capture microdissection combined with microarray analysis allowed for the determination of the transcriptional signature of scarce cells in fish gills, such as ionocytes, and aided characterization of the transcriptome of these cells in freshwater and seawater acclimated trout.

## Introduction

Fish gills have several functions implicated in the maintenance of ion and gas homeostasis (respiration, osmoregulation, acid-base regulation, and nitrogen secretion) [1]. Due to direct contact with the external medium and the possibility of contact with pollutants or pathogens, the fish gill also has a barrier role and presents certain mechanisms of xenobiotic biotransformation [2] and immune defense [3]. Thus, to accomplish all these functions, gill morphology is very complex. This organ is composed of filaments and lamellae covered by epithelia and supported by cartilage and pillar cells. Epithelia are subdivided into two regions: a primary epithelium covering the filament and a secondary epithelium covering the lamellae [4].

Epithelia are composed of two epithelial cell types directly in contact with the external medium, pavement cells and mitochondria-rich cells (MRCs), representing more than 90% and less than 10% of the epithelial surface area, respectively [1]. Scarce mucous cells were also observed in contact with the external medium. Under these epithelial cells, both undifferentiated and basal cells have been characterized [5].

MRCs, more recently named ionocytes, are a very important cell type implicated in ion transport to maintain fish blood homeostasis. Ionocytes absorb and secrete NaCl in freshwater (FW) and saltwater (SW) environments, respectively. These cells are also implicated in Ca^2+^ absorption, H^+^/HCO_3_^-^ flux for acid-base regulation and ammonia excretion. In fresh water, several ionocyte sub-types have been identified in different fish species [6]. These sub-types were characterized using ultrastructural morphology and/or several cell biology and physiological approaches [7–9]. In contrast, only one sub-type has been identified in seawater fish species [7–9]. This SW subtype is closely associated with the accessory cells that send out digitations within the apical part of the ionocytes. In salmonids, two FW ionocyte sub-types were identified using (i) ultrastructural studies, where α-ionocytes and β-ionocytes were observed at the base of the lamellae and in the interlamellar region, respectively [10], (ii) density gradient separation techniques combined with differential peanut lectin agglutinin (PNA) staining to identify PNA+ and PNA- cells [11] and (iii) triple-color immunofluorescence staining for NKA, NKCC1 and NHE3b [12].

For euryhaline fish, an important gill remodeling process occurs after transfer from fresh water to sea water or brackish water. The gill epithelium is transformed from a salt absorbing to a salt secreting epithelium. To understand salinity adaptation, several studies have performed large scale gene expression experiments on gill tissue in *Dicentrarchus labrax* [13], *Anguilla anguilla* [14], *Anguilla japonica* [15], *Gillichthys mirabilis* [16], *Sarotherodon melanotheron* [17], *Fundulus heteroclitus* [18]. These studies led to the characterization of important gill osmotic effectors and signaling pathways that develop during adaptation to osmotic stress. However, because ionocytes are scarce in gill tissue, the expression of genes specific to this cell type can be hidden by the global gene expression of fish gill cells. Several studies performed gill dissociation and ionocyte separation for candidate gene expression and proteomic approaches of eel MRCs [19–21]. However, no large-scale gene expression study has been conducted on these dissociated cells. To develop such a goal, we elected to develop a new approach that aimed to isolate gill ionocytes directly from tissue using laser capture microdissection (LCM) [3,22]. In contrast to cell dissociation, the LCM approach is not stressful for cells. This technique has become a valuable method for isolating individual cell types from heterogeneous tissues [23]. Preliminary studies have used laser capture microdissection to isolate intraepithelial lymphoid tissue from gills and revealed the expression of T-cell receptor transcripts in this area [3]. Combining LCM with microarray technology has the potential to identify the transcriptomic signature of specific cells. In this study, our objective was to compare the transcriptome signature of freshwater and seawater ionocytes using laser capture microdissection and microarray approach.

## Materials and Methods

### Ethics statement

Experimental research performed in this study was in accordance to the guiding principles for the use and care of laboratory animals and in compliance with the French and European regulations on animal welfare. Experimenters obtained authorization from the French Government to carry out animal experiments (Agreement No. 35–57 for IL). Experiments were conducted within INRA facilities that had authorization for animal experimentation (B29-777-02 and B35-238-6) and were approved by the Local Animal Care and Ethic Committee of INRA (approval n° B9029).

### Fish conditioning and sampling

Immature rainbow trout (*Oncorhynchus mykiss*) bred in a local hatchery (Drennec, Sizun, France) were transferred to the laboratory facilities. After acclimation to the tanks for at least 1 week, the fish were directly transferred from fresh water to either fresh water (control group) or 35 ppt sea water. The protocol to transfer fish to SW was previously described [24]. The trout were maintained in a recirculated water system at 10–13°C under a natural photoperiod and fed commercially available pellets (BioMar, Nessac, France). Fish (90–140 g), kept either in fresh water or transferred into sea water, were collected at various time points following the salinity change. After euthanized with a lethal dose of phenoxyethanol, blood and gill tissue were sampled. Plasma was stored at -20°C until further use. The second gill arches were collected for laser capture microdissection (LCM) and in situ hybridization (ISH). For LCM, the gill arches were frozen in isopentane by submersion in liquid nitrogen and storage at -80°C until further use. For ISH, the gill arches were fixed for 8 hours at 4°C with 4% paraformaldehyde in phosphate buffered saline, then gradually dehydrated in methanol and stored in 100% methanol at −20°C.

### Plasma parameters

Plasma sodium was analyzed using a model 410 flame photometer (Sherwood Scientific). Chloride and calcium were assessed using colorimetric kits (chloride with a mercuric-thiocyanate method and calcium with the Arsenazo III method (Biolabo, Maizy, France)). The values reported in the figures are mean ± s.e.m. The Mann-Whitney U-test was used to make comparison between freshwater and seawater at each time point after non-parametric analysis of variance (Kruskal-Wallis test). All these statistical analyses were done with Statistica software (Statsoft, Maisons-Alfort, France). Differences were considered as significant when P<0.05.

### Fluorescent immunohistochemistry with Zenon labeling technique

Gill cryosections (10 μm thick with a Leica cryostat) were cut, mounted onto super frost glass slides and processed for immunostaining with Na/K-ATPase antibody (α5 antibody) to label the ionocytes. To reduce the quantity of incubations and washes, the antibody was conjugated with Zenon fragment. A mouse IgG labeling kit (Zenon Alexa Fluor 488, Molecular Probes, Life Technologies, Saint Aubin, France) was used, and the IgG labeling was performed according to the Zenon Complex Formation protocol and diluted, in PBS with RNase inhibitor, to the desired working concentration. After incubation with the labeling complex for 5 min at 4°C, the sections were washed with PBS-RNAse inhibitor, dehydrated (70% ethanol, 95% ethanol (2 x 30 sec), 100% ethanol (3 x 30 sec) and xylene (3 x 1 min)) and then air dried (2 min) before LCM. The mouse monoclonal anti- chicken Na/K-ATPase (α5 antibody) developed by Douglas M. Fambrough was obtained from the Developmental Studies Hybridoma Bank developed under the auspices of the NICHD and maintained by The University of Iowa, Department of Biological sciences, Iowa City, IA 52242.

### Laser capture microdissection

Arcturus® Veritas (Applied Biosystems, Life technologies, Saint Aubin, France) was used to capture fluorescently labeled ionocytes. The infrared laser was adjusted to produce spot sizes between 10 and 15 μm in diameter, which allowed consistent capture of cells onto CapSure LCM caps (Excilone, Elancourt, France). The cells on the caps were lysed (lysis buffer from the PicoPure® RNA isolation kit (Excilone, Elancourt, France)) for RNA extraction. To limit RNA degradation, the time between cell labeling and lysis was routinely no longer than 1 hour. During this time, a mean of 400 labeled ionocytes can be selected and captured from each animal. Ionocytes from four freshwater and four seawater trout were captured for this study.

### RNA extraction and quality control

RNA was extracted using the PicoPure RNA isolation kit (Excilone) following the manufacturer’s recommendations and with 1 U of DNase (Invitrogen, Life Technologies, Saint Aubin, France) treatment for 15 min at room temperature. RNA was extracted from the gill sections and the ionocytes trapped in the CapSure LCM caps. The quality and purity of the gill section RNA were evaluated after cutting the frozen gill section. RNA quality was assessed by an Agilent 2100 Bioanalyzer using the RNA 6000 pico LabChip kit (Agilent Technologies, Massy, France).

### RNA amplification

Ionocyte RNA from four freshwater and four seawater trout was amplified and purified using the Ovation picoSL WTA system V2 RNA Amplification kit (NuGEN Technologies, Leek, Netherlands) according to the manufacturer’s instructions. Linear amplification was obtained by reverse transcription of the RNA by a cDNA tag with a SPIA sequence. This amplification used a DNA/RNA chimeric primer, DNA polymerase and RNase H. RNase H removes the RNA portion of the heteroduplex SPIA tag sequence, revealing a binding site for the SPIA primer. The process of SPIA DNA/RNA primer binding, DNA replication, strand displacement and RNA cleavage is repeated in a processive manner. Amplified cDNA was quantified using a NanoDrop Spectrophotometer (Labtech, Palaiseau, France).

### Microarray analysis

Ionocyte RNA from four individual freshwater and four individual seawater trout (biological replicates) was used for microarray analysis. Rainbow trout RNA expression profiling was conducted using an Agilent 8x60K high-density oligonucleotide microarray (GEO platform #GPL15840).

The one color labeling and hybridization steps of the amplified cDNA were performed according to the “Gene Expression FFPE Workflow” Agilent protocol (starting from step 5 with minor modifications). Briefly, 1.65 μg of amplified cDNA was labeled using the Genomic DNA ULS Labeling Kit (1 μl of ULS-Cy5 per μg of DNA) at a final volume of 20 μl. The labeled cDNA was then purified using an Agilent KREApure column according to the standard protocol. The yield (~100%) and the degree of labeling (between 1.5% and 3%) of Cy5-cDNA were checked in all samples using a Nanodrop ND–1000 spectrophotometer (Labtech, Palaiseau, France). Cy5-cDNA (600 ng) was denatured at 95°C in a final volume of 34 μl with Agilent 100X Blocking Agent and 2X Hi-RPM GE Hyb buffer. After adding 11 μl of Agilent-CGHblock, the labeled cDNA was finally hybridized on a sub-array. Hybridization was performed for 17 hours at 65°C in a rotating hybridization oven (20 RPM) prior to washing and scanning with an Agilent Scanner (Agilent DNA Microarray Scanner, Agilent Technologies, Massy, France) using the standard parameters for a gene expression 8x60K oligoarray (3 μm and 20 bits). Data were then obtained with the Agilent Feature Extraction software (10.7.1.1) according to the appropriate GE protocol (GE1_107_Sep09). Normalization and statistical analysis were performed using GeneSpring software (Agilent). Data were scale-normalized using the median value of each array, then log_2_ transformed before statistical analysis. Spots not significantly different from background were excluded from analysis. Probes were considered valid when corresponding spots remained present in at least 75% of the replicates of each experimental condition after the flagging procedure. To identify differentially expressed genes between gill ionocytes from trout maintained in fresh water (4 individual biological replicates) or acclimated to sea water (4 individual biological replicates), a T-test with a Benjamini-Hochberg correction (p-value<0.05) was performed on a set of genes previously selected with a Fold Change>3. Clustering analysis was performed and visualized using CLUSTER and TREEVIEW software [25]. For clustering, the data were log transformed, median-centered and an average linkage clustering was carried out. Microarray data were submitted to the GEO database with the reference GSE69409.

### *In situ* hybridization and immunohistochemistry

The following bacterial clones were obtained from the USDA (Washington, DC, USA): 1RT64K03_A_F02 (SLC26A6), 1RT103M12 (Clcn2), and 1RT129G21_A_D11 (NBC). The bacteria containing plasmids were grown in LB-ampicillin medium. Plasmids were extracted (NucleoSpin Plasmid kit, Macherey-Nagel, Hoerdt, France) and cDNA inserts were amplified by PCR using vector-specific primers (Sigma), Go Taq DNA polymerase, and dNTPs (Promega). The PCR products were used as templates for digoxigenin-labeled probe synthesis using digoxigenin-conjugated UTP (Roche Diagnostics Corp,Meylan, France) and T7 or SP6 RNA polymerase (Promega, Harbonnières, France) for the antisense (specific signal) or sense (negative control) RNA probes.

In situ hybridization experiments were performed on the gill filaments. After fixation of the gill arches in 4% paraformaldehyde, the gill filaments, stored at -20°C in methanol, were cut above the gill septum, placed in baskets and deposited inside "In situ Pro VS automate" (Intavis Bioanalytic Instruments) to perform in situ hybridization using the following conditions. Briefly, the filaments were rehydrated in graded methanol-PBS, pre-hybridized for 1 hour at 65°C in hybridization solution (50% formamide, 5x SSC, 0.1% Tween20, 0.005% heparin, and 0.1 mg/ml tRNA) and hybridized for 16 hours at 65°C with 0.5–1 ng/μl of DIG-labeled cRNA probes. After several post-hybridization washes with hybridization solution at 65°C, then SSC/Tween20 solutions at 55°C, the gill filaments were incubated in blocking buffer (PBS, 0.2% TritonX100, 0.2% Tween20, and 2% serum) for 1 hour at room temperature. Then, the filaments were incubated with anti-DIG antibody coupled to alkaline phosphatase (1:1000 Roche Diagnostics Corp, Meylan, France) in blocking buffer for 6 hours. After several washes in PBS/Tween20 and detection buffer (100 mM Tris 1 M pH 9.5, 100 mM NaCl M, 50 mM MgCl_2_ 1 M pH 9), the color reaction was performed in the presence of NBT/BCIP in detection buffer (Roche Diagnostics Corp, Meylan, France). After several PBS washes, post fixation in 4% paraformaldehyde in PBS and several rinses, the gill filaments were dehydrated and embedded in paraffin. The tissue samples were sectioned at 5 μm. All hybridizations were performed on several filaments of at least 3 fish per condition.

To identify the ionocytes, immunohistochemistry was performed on the gill filament sections using the UltraVision Kit and Labvision components (Microm France, Francheville, France). Briefly, deparaffined and rehydrated sections were treated for at least 5 min in 0.01 M PBS + 0.05% Tween20 (PBS-Tween20). The sections were incubated for 10–15 min in a hydrogen peroxide block to reduce endogenous peroxidase activity. After two washes in PBS-Tween20, the slides were then incubated for 5–10 min in Ultra V Block to block non-specific background staining. After one rinse with PBS, anti-Na/K-ATPase monoclonal antibody was applied overnight in a humidity chamber at 4°C. After 4 washes with PBS-Tween20, the sections were incubated for 10 min at room temperature with biotinylated secondary antibody (goat anti-polyvalent: anti-mouse and anti-rabbit) followed by 4 rinses and 10 min of incubation with a streptavidin peroxidase complex at room temperature. The chromogene selected for labeling was DAB (3,3’-diaminobenzidine), chosen according to manufacturer recommendations. The anti-Na/KATPase (α5 antibody) developed by Douglas M. Fambrough was obtained from the Developmental Studies Hybridoma Bank developed under the auspices of the NICHD and maintained by The University of Iowa, Department of Biological sciences, Iowa City, IA 52242.

### Quantitative real-time PCR

qPCR was performed on a StepOnePlus real-time PCR system (Applied Biosystems, Life Technologies). Reactions were performed in duplicate and in a total volume of 10 μl with 4 μl of diluted cDNA, 5 μl of Fast SYBR Green Master Mix (Applied Biosystems, Life Technologies) and 200–400 nM of each primer (see Table 1 for the list of primers). The RNA expression level was normalized by Fyn (clone 1rt99c24_c_b12, GenBank CA357459, Proto-oncogene tyrosine-protein kinase FYN (FYN)). Fyn was chosen because of its invariant expression in previous microarray experiments comparing freshwater and seawater gill transcriptomes. Primers targeting fyn (GenBank CA357459) and SLC26A6 (CA357980.p.om.8, www.sigenae.org) were designed using Primer3 software [26]. For Na/K-ATPase α1a, α1b and NaHCO3, we used primers already designed in [27,28]. A melting curve was generated to confirm product specificity. Expression data were calculated by the 2^−ΔΔCt^ method. The values reported in the figures are mean ± s.e.m. Statistical differences between FW and SW ionocytes were examined by Mann-Whitney U-test using Statistica software (Statsoft, Maisons-Alfort, France)

**Table 1:** Primer pairs for real time quantitative RT-PCR.

Gene name	Forward primer sequences	Reverse primer sequences
Na/K-ATPase α1a	GCAGACGCCTCTCGGAATT	CAATGAGAAAGATGATGGATGG
Na/K-ATPase α1b	GGAAGACGCCTATAGCCAAA	CGATGAGGAAGATGACAGCTTC
NBC	TGGACCTGTTCTGGGTAGCAA	AGCACTGGGTCTCCATCTTCAG
SLC26A6	CTAAAGCCTCCCAGTTCACC	AGACCAACAGCCACCATCTC
FYN	CCGAGCACAGATAGGAGGAG	CACGCACACAGACACAAGTG

## Results and Discussion

**Fig 1:**
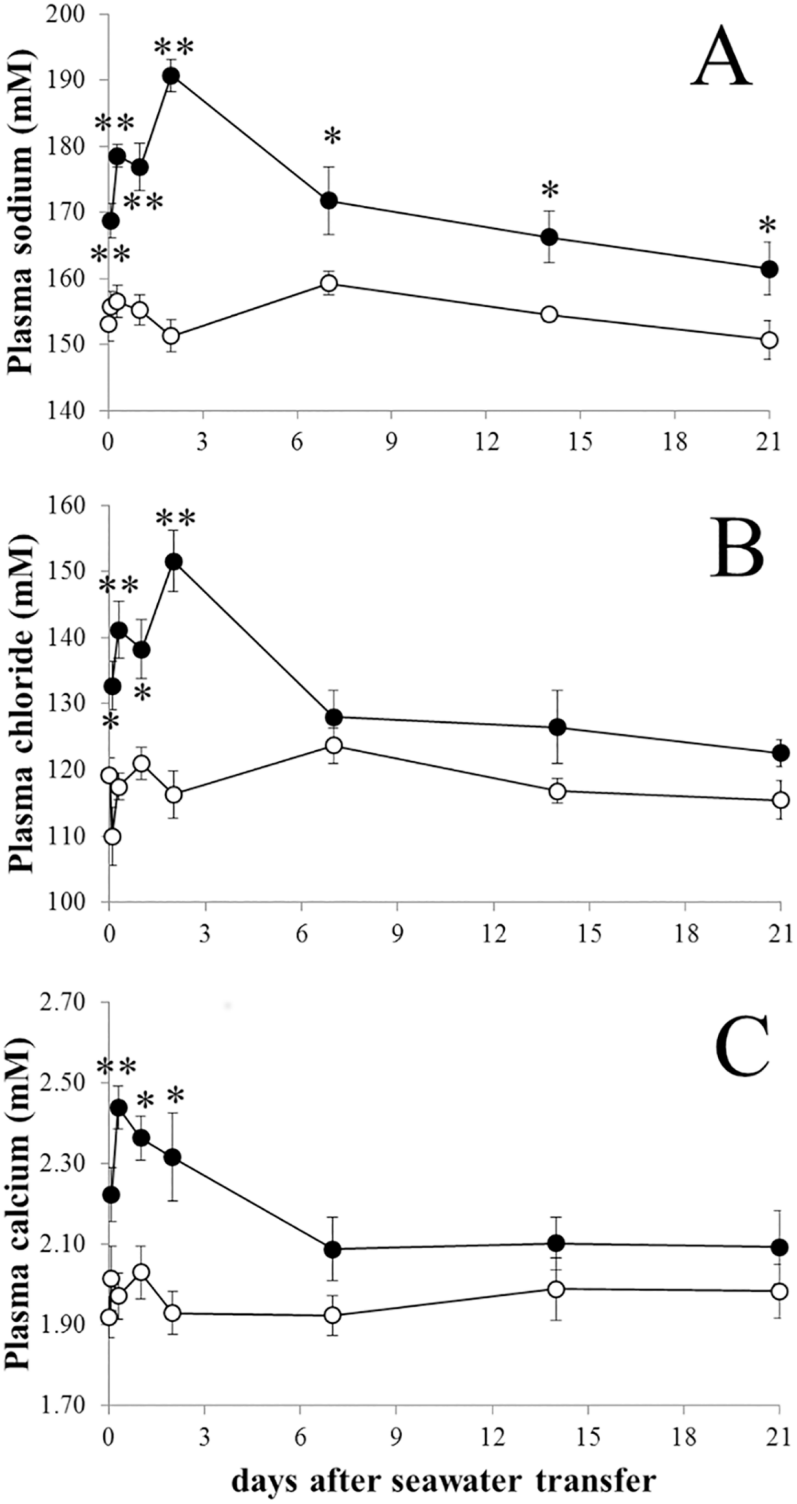
Plasma ion concentrations after sea water transfer. Mean plasma sodium (A), chloride (B), and calcium (C) from time 0 to 21 days post sea water transfer. Open and filled circles represent respectively freshwater and seawater transferred trout. Values represent means ± s.e.m. of six fishes. Differences between freshwater and seawater at each time point were assessed with non-parametric Mann-Whitney U-test after non-parametric analysis of variance (Kruskal-Wallis test). * and **, significantly different from the corresponding values in freshwater at P<0.05 and P<0.01.

The transfer of rainbow trout from fresh water to sea water induced a transient increase of plasma sodium, chloride and calcium, followed by the return of chloride and calcium to basal levels and a plateau to near freshwater plasma concentration for sodium (Fig 1). In accordance with a previous study [29], our results indicated that after 14 days in sea water, trout were able to regulate their hydromineral balance. Consequently, transcriptome analysis of ionocytes was compared between fish transferred for 14 days in seawater or freshwater tanks.

To compare ionocyte transcriptomes in both FW and SW, an immuno-laser capture microdissection was performed on the trout gill sections. Because the Na/K-ATPase pump (NKA) is highly expressed in ionocytes, whatever the salinity, an NKA antibody against the α-subunits was used as a marker for identifying ionocytes on the tissue sections.

**Fig 2:**
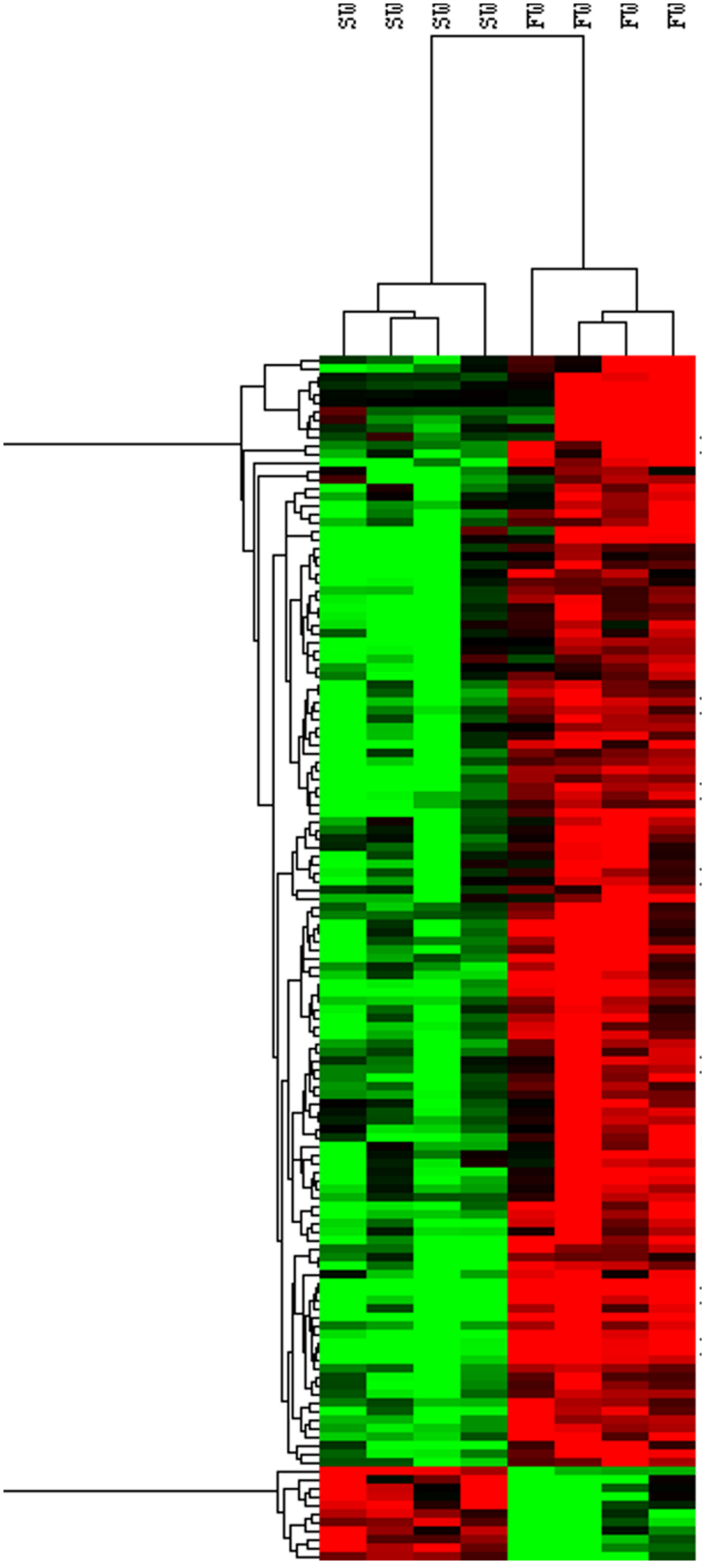
Hierarchical clustering analysis of gene differentially expressed between FW and SW ionocytes. Analysis was performed based on a log_2_-transformed ratio value of 138 genes differentially expressed. Row and columns represent genes and samples respectively. Expression levels of log_2_-transformed ratio are represented by a color tag: red and green for high and low levels of expression respectively.

Using the selected genes with a fold change >3 between freshwater and seawater ionocytes as well as a t-test with a Benjamini-Hochberg correction (p-value<0.05), 138 genes appeared differentially expressed. Among these genes, 127 were up and 11 down-regulated in freshwater compared to seawater ionocytes (Fig 2 and S1 Table). The genes up-regulated in FW ionocytes corresponded to 98 annotated genes (certain genes were represented by several oligos, such as SLC4A4 (5 probes); S1 Table) and 14 non-annotated genes. In seawater ionocytes, among the 11 oligonucleotides with higher expression, 10 corresponded to annotated genes.

Interestingly, when comparing RNA expression profiles between FW and SW ionocytes, most genes appeared up-regulated in FW ionocytes. Gene expression in other species did not present such difference in gill expression between FW and SW acclimated fish [13,14]. However, in these studies, RNA expression of whole gill tissue was used to perform transcriptome analysis. In our study, we attained transcript expression of one cell type present in less than 10% of whole gill tissue.

Interestingly, the most differentially expressed genes (fold change >9) were ionic transporters (S1 Table). This is consistent with the role of ionocytes in ion transport (FW and SW ionocytes absorb and secrete salt, respectively). Other genes; grouped according to their functions using uniprot (http://uniprot.org), gene ontology, literature; revealed more abundant genes involved in structural organization and regulation in FW ionocytes (Table 2). Some of these genes were implicated not only in extracellular matrix composition and cytoskeleton organization but also in cell adhesion and motility. Another set of genes was grouped into biological functions related to metabolism. The first group was related to energetic metabolism, implicating several mitochondrial enzymes in ATP synthesis, and the second group was principally involved in amino acid and protein metabolism. Finally, several genes implicated in transcriptional regulation, cell signaling and the cell cycle were also expressed more in FW ionocytes.

**Table 2:** Annotated genes exhibiting differential expression between FW and SW ionocytes.

Gene identification	function
Sodium/potassium-transporting ATPase subunit alpha–1	ion transport
Chloride channel protein 2	ion transport
Solute carrier family 26 member 6	ion transport
Electrogenic sodium bicarbonate cotransporter 1	ion transport
Sodium/potassium-transporting ATPase subunit beta–233	ion transport
Zinc transporter	ion transport
P3 protein	ion transport
Major facilitator superfamily domain-containing protein 1	transport
Collagen alpha–1(X)	extracellular matrix
Collagen alpha–1(I) chain	extracellular matrix
Collagen alpha–2(I) chain	extracellular matrix
Fibronectin	extracellular matrix
Sparc	extracellular matrix
sparc/osteonectin	extracellular matrix
Bridging integrator 3	cytoskeleton
Coronin–6	cytoskeleton
Cytoplasmic dynein 1 intermediate chain 2	cytoskeleton
Sorting nexin–3	cytoskeleton
Spastin	cytoskeleton
Transgelin	cytoskeleton
Tubulin alpha-1D chain	cytoskeleton
Vesicle-associated membrane protein 5	cytoskeleton
*Uncharacterized protein ENSP00000361571*	*cytoskeleton*
CD9 protein	cell adhesion
Carcinoembryonic antigen-related cell adhesion molecule 5	cell adhesion
Liprin-beta–1	cell adhesion
Vascular cell adhesion protein 1	cell adhesion
Claudin–4	junction
Peripheral myelin protein 22	junction
Isocitrate dehydrogenase [NADP], mitochondrial	energetic metabolism
NADH dehydrogenase [ubiquinone] flavoprotein 1, mitochondrial	energetic metabolism
NADH dehydrogenase [ubiquinone] flavoprotein 2, mitochondrial	energetic metabolism
Cytochrome b-c1 complex subunit 6, mitochondrial	energetic metabolism
Up-regulated during skeletal muscle growth protein 5	energetic metabolism
Aconitate hydratase, mitochondrial	energetic metabolism
Aldehyde dehydrogenase, mitochondrial	response to oxygen level
Hemoglobin subunit beta–1	response to oxygen level
NAD(P) transhydrogenase, mitochondrial	response to oxygen level
*Selenoprotein M*	*response to oxygen level*
5-methyltetrahydropteroyltriglutamate–-homocysteine methyltransferase	amino acid synthesis
Ornithine decarboxylase	amino acid synthesis
Proline synthetase co-transcribed bacterial homolog protein	amino acid synthesis
Fetuin-B	protein metabolism
Serine protease inhibitor Kazal-type 2	protein metabolism
ATP-binding cassette sub-family F member 1	protein metabolism
COP9 signalosome complex subunit 5	protein metabolism
DCN1-like protein 4	protein metabolism
Eukaryotic translation initiation factor 4E-1A	protein metabolism
Proteasome subunit beta type–7	protein metabolism
26S proteasome non-ATPase regulatory subunit 5	protein metabolism
Uridine 5-monophosphate synthase	protein metabolism
RING finger protein 152	protein metabolism
Transmembrane protease serine 9	protein metabolism
NEDD8-conjugating enzyme UBE2F	protein metabolism
Ubiquilin–4	protein metabolism
*Eukaryotic translation initiation factor 3 subunit B*	*protein metabolism*
*40S ribosomal protein S7*	*protein metabolism*
Glycerol-3-phosphate dehydrogenase 1-like protein	Carbohydrate-lipid metabolism
Arylsulfatase B	glucopolysaccharide catabolism
Glycogen phosphorylase, muscle form	glycogen metabolism
Elongation of very long chain fatty acids protein 1	lipid metabolism
Putative phospholipase B-like 1	lipid metabolism
Splicing factor, arginine/serine-rich 6	mRNA splicing
U1 small nuclear ribonucleoprotein A	mRNA splicing
Exosome complex exonuclease RRP44	RNA catabolic process
Eukaryotic peptide chain release factor subunit 1	RNA catabolic process
Early growth response protein 1	regulation of transcription
far upstream element (FUSE) binding protein 1	regulation of transcription
GON-4-like protein	regulation of transcription
Histone deacetylase 1	regulation of transcription
Hepatic leukemia factor	regulation of transcription
LIM/homeobox protein Lhx6	regulation of transcription
Protein LLP homolog	regulation of transcription
Protein max	regulation of transcription
Polycomb protein SCMH1	regulation of transcription
TSC22 domain family protein 3	regulation of transcription
*Forkhead box protein N3*	*regulation of transcription*
*Histone acetyltransferase MYST2*	*regulation of transcription*
*Thioredoxin-related transmembrane protein 1*	*regulation of transcription*
C4b-binding protein alpha chain	immune system
Putative HLA class I histocompatibility antigen, alpha chain H	immune system
T-cell immunomodulatory protein (Fragment)	immune system
*T-cell receptor beta chain C region*	*immune system*
Dolichyl-diphosphooligosaccharide-protein glycosyltransferase subunit	cell cycle_anti-apoptosis
Probable Bax inhibitor 1	cell cycle_anti-apoptosis
RNA-binding protein 24	cell cycle_differentiation
cAMP-regulated phosphoprotein 19	cell cycle_division
Salmo salar Gametogenetin-binding protein 2	cell cycle_division
Guanine nucleotide-binding protein G(i) subunit alpha–2	cell cycle_division
Protein NDRG3	cell cycle-division
Heme-binding protein 2	cell cycle_pro-apoptosis
Frizzled–1	cell signaling
Protein Wnt–11	cell signaling
Growth factor receptor-bound protein 2	cell signaling
Homer protein homolog 2	cell signaling
Hepatocyte growth factor receptor	cell signaling
3-phosphoinositide-dependent protein kinase 1	cell signaling
Gamma-aminobutyric acid receptor-associated protein-like 2	cell signaling
*Arylamine N-acetyltransferase*, *pineal gland isozyme NAT–10*	*detoxification*
Post-GPI attachment to proteins factor 3	GPI anchor metabolic process
Metalloreductase STEAP3	iron homeostasis
Transposable element Tcb2 transposase	DNA integration

Genes in italic were upregulated in SW and others genes were upregulated in FW.

### Transporters

Because ionocytes are the major cell type implicated in transepithelial ion transport in the gill, the first genes examined in the list of differentially expressed genes between freshwater and seawater ionocytes were the ion transporters. Indeed, certain transporters appeared up-regulated in freshwater ionocytes.

**Fig 3:**
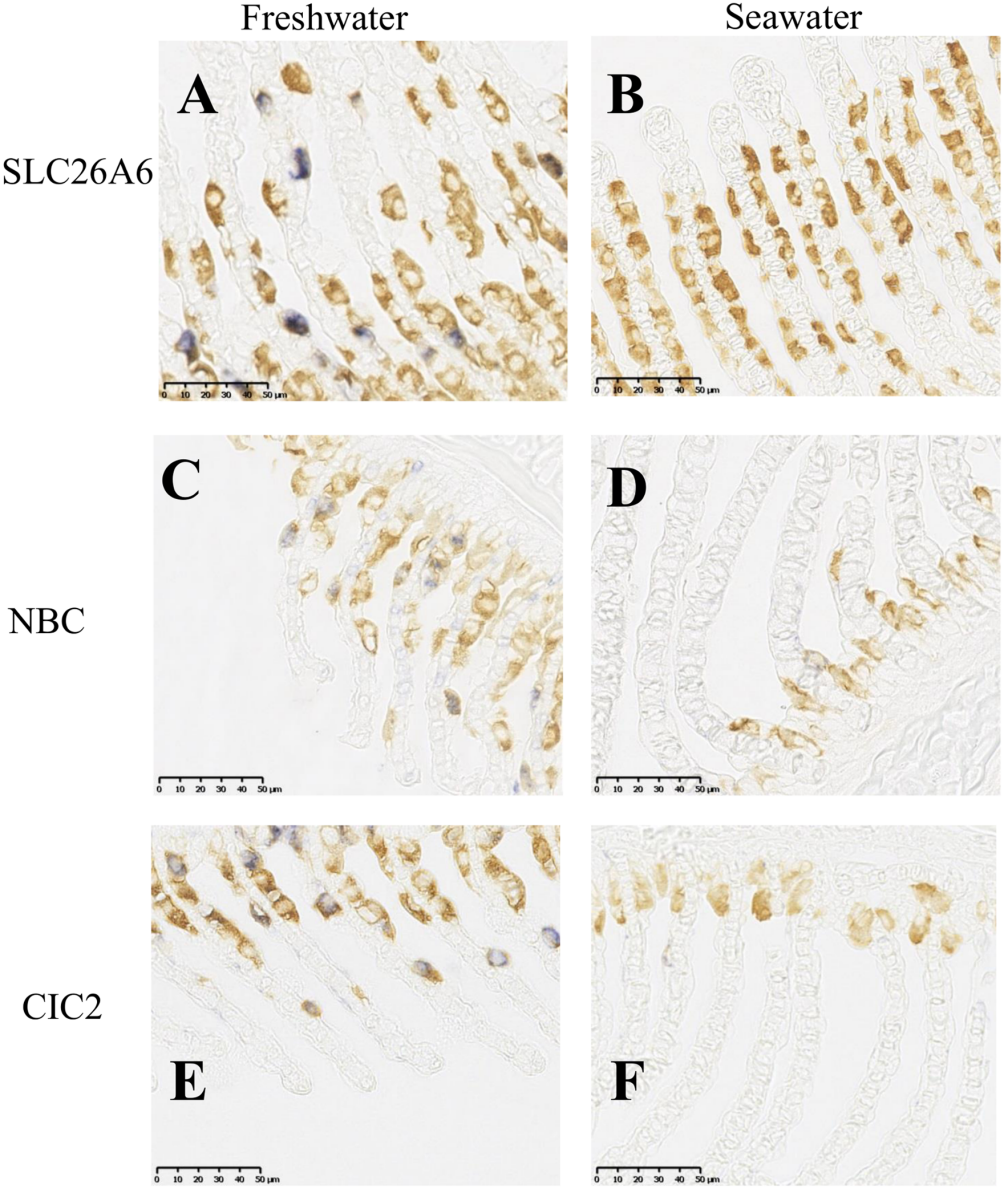
*In situ* hybridization of 3 genes (purple labelling) in fish gill from freshwater (A, C, E) or seawater (B, D, F) acclimated rainbow trout associated with immunocytochemistry of ionocyte with Na/K-ATPase antibody (brown labelling). AB: SLC26A6, CD: NBC, EF: CIC2.

One of the most interesting pieces of information provided by this transcriptomic analysis is related to chloride channel 2 (CIC2), which was expressed more in freshwater ionocytes in our microarray analysis. Interestingly, in situ hybridization (i) confirms this result with no detectable CIC2 mRNA in the SW ionocytes and (ii) shows expression only in FW ionocytes with no detectable labeling in pavement cells or other gill cells (Fig 3). CIC2 is an anion channel located in the surface membranes of excitable and epithelial mammal cells [30,31]. One important function of CIC2 is chloride efflux at the basolateral membrane in the gastro-intestinal tract of guinea pigs [32,33]. Our results suggest that CIC2 acts as a channel participating in chloride absorption in FW rainbow trout gills and more particularly in basolateral flux from cells to the blood. In accordance with our results, two studies on zebrafish revealed the presence of CLC-2c in ionocytes [34,35]. CIC3 was previously reported to be a good candidate for involvement in basolateral chloride transport at the level of the gill epithelium in other teleost fish species, *Oreochromis mossambicus* [36,37], *Tetraodon nigroviridis* [38] and *Dicentrarchus labrax* [39]. However, in our study, the RNA expression of CIC3 in the trout microarray was not significantly different between FW and SW ionocytes. Overall, our data suggest that basolateral chloride transport in the FW ionocytes of rainbow trout is most likely associated with CIC2 as in zebrafish, and not CIC3, as in other fish species.

**Fig 4:**
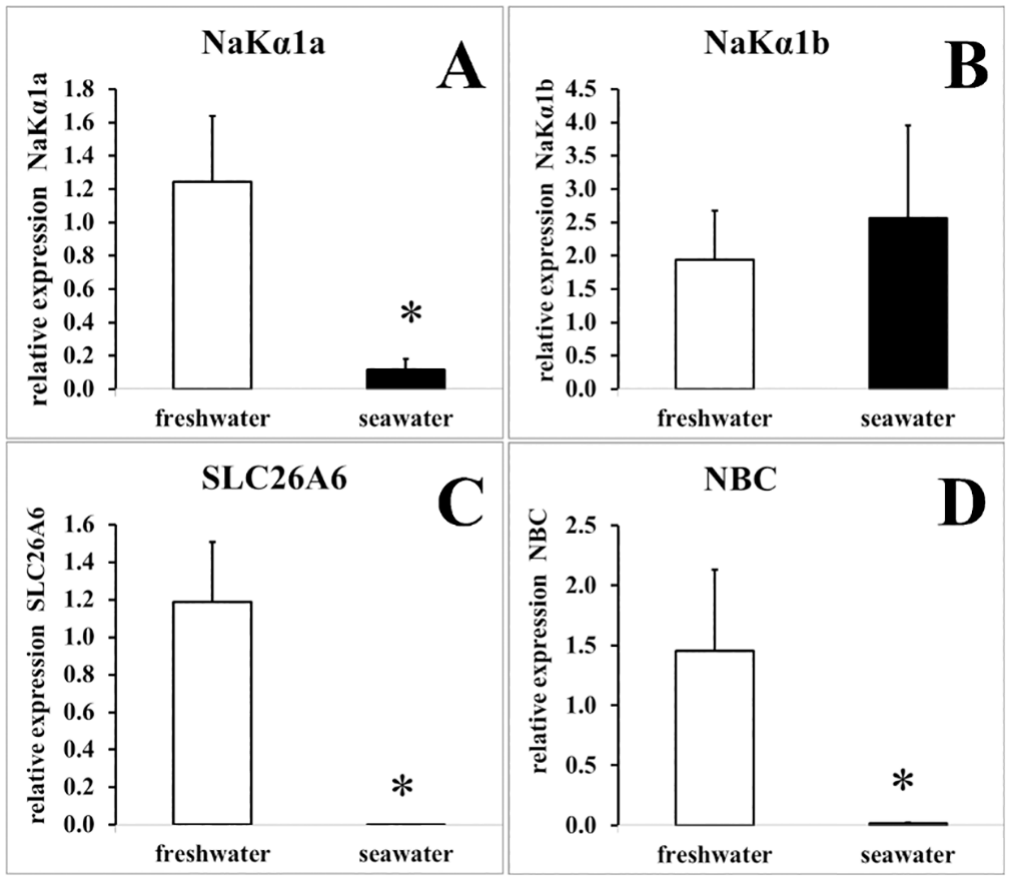
Quantitative real-time PCR of 4 genes in freshwater and seawater ionocytes. Values represent means ± s.e.m. of four fishes. *, significantly different from the corresponding values in freshwater at P<0.05, Mann-Whitney U-test.

The present transcriptomic analysis also indicated that another chloride transporter, SLC26A6 (a Cl-/HCO_3_ exchanger from the SLC26 family), had higher mRNA expression in freshwater ionocytes. This result was obtained using a microarray experiment and validated with qPCR (Fig 4C). Furthermore, in situ hybridization showed expression of this exchanger only in freshwater ionocytes (Fig 3). Our results corroborated another recent study showing higher expression of this transporter in the gills of rainbow trout in fresh water compared to salt water [40]. Overall, using complementary approaches, the present study allowed clear identification of SLC26A6 in ionocytes with no detectable labeling in pavement cells or other freshwater gill cells.

Expression analysis of the different subunits of the Na/K-ATPase pump, which provides the driving force to absorb (in FW) and secrete (in SW) NaCl, also provided interesting information. Among the subunits of the Na/K-ATPase pump, the expression levels of the α1a isoform and one β-subtype (NKAβ_233_, the duplicate copy of NKA-β_1_ in *Anguilla anguilla*) were higher in FW than in SW ionocytes (Table 2 and Fig 4A). The co-expression of NKA α1a and this β_1_-subtype suggests their link to Na/K-ATPase activity in freshwater ionocytes. The pump consists of three subunits, α, β, and γ [41]. Among the α-subtypes, α1a and α1b were significantly decreased and increased, respectively, in salmonid gills after transfer from FW to SW [42–44]. In accordance with previous studies using whole gill tissue, the transcript expression of the Na/K-ATPase α1a isoform was significantly reduced in seawater ionocytes in the present study (Fig 4A). In contrast with these previous studies, our data showed that mRNA expression of isoform α1b in isolated ionocytes was not significantly different between FW and SW, neither in microarray nor in qPCR analysis (Fig 4B). This difference in transcript expression between whole gill tissue and ionocytes suggests either a higher number of ionocytes in SW versus FW or higher RNA expression of this α1b isoform in other gill cell types.

For gill sodium flux in rainbow trout, several ion transporters were proposed in FW fish [6]. One model implicated a Na^+^ channel and a Na^+^/HCO_3_^-^ cotransporter (NBC) in the apical and basolateral side of ionocytes, respectively [45]. Our results confirm the involvement of a NBC (SLC4A4) in trout gill ionocytes (microarray in Table 2, ISH in Fig 3, and qPCR in Fig 4D). Furthermore, this co-transporter appears to be predominantly involved in freshwater environments, as gill mRNA expression is significantly reduced after at least 2 weeks in sea water. Our results are in agreement with others studies showing NBC in FW ionocytes of Osorezan dace [46], zebrafish [47] and Mozambique tilapia [48]. No sodium channel isoforms were differentially expressed in our microarray experiment. Recently, members to the amiloride-sensitive ENaC/degenerin family of ion channel were identified as putative Na^+^ channels involved in apical sodium transport in rainbow trout gill cells: acid-sensitive channels (ASICs) [49]. However the two trout subunits of ASICs identified in gill are not present on our microarray, further experiments will be necessary to compare their gene expression between FW and SW ionocyte. Interestingly, several Epithelial Na^+^ Channel (ENaC) regulators, identified in other animals, appeared up-regulated in FW ionocytes in this study. Thus, we observed an increase in "sorting nexin3" RNA expression, which encodes a protein known to increase the cell surface expression of ENaCs [50]. Another gene, TSC22 domain family protein 3 (or Glucocorticoid-Induced Leucine Zipper protein) has been shown to stimulate ENaC cell surface expression and activity [51]; in the present study, we observed an increase in its expression in FW ionocytes. Overall, these data do not demonstrate the presence of an active epithelial Na^+^ channel but instead show possible involvement of regulatory pathways related to that channel in FW.

Other transporters that were enhanced in FW ionocytes included two solute carrier proteins (P3 protein (SLC10A3) and a zinc transporter (SLC39A11)). Solute carrier family 10 comprises influx transporters for various molecules, such as bile acids, steroidal hormones and drugs [52]. SLC10A3 exhibits amino acid identity with other SLC10 proteins, however its function and substrate specificity are not yet known [52]. SLC39A11 is a zinc transporter from the ZIP family. This family increases the intracellular zinc concentration via uptake across the plasma membrane or efflux from intracellular compartments [53]. Higher SLC39A11 expression in FW ionocytes suggests an important role of zinc in certain cellular processes. Another transporter, MFSD1 (major facilitator superfamily domain-containing protein 1), was also up-regulated in FW. However, like SLC10A3, the function and solute transported are unknown. Further investigation is necessary to characterize the role and importance of each of these transporters in ionocyte functions.

### Extracellular matrix (ECM) and structural cellular proteins

Interestingly, several genes related to cell structure and morphogenesis (extracellular matrix and cytoskeleton) presented a higher expression level in FW ionocytes than in SW ionocytes (Table 2). This is not unexpected, as up-regulation of these expression transcripts can not only be related to differences in the morphological ultrastructure of ionocytes in fresh water and sea water [8,10] but also to their localization. In FW, ionocytes are present in the lamellae and filament, whereas in SW, most ionocytes are located in the filament. Thus, the morphology and localization of the ionocytes are different between FW and SW, which may be associated with the contrasting transcriptomic signatures of structural proteins in FW and SW ionocytes.

Among the structural genes, several extracellular matrix component proteins were up-regulated in FW ionocytes, including collagens (COL1A1, COL1A2, and COL10A1) and fibronectin (FN1). The RNA expression of two proteins associated with the extracellular matrix was also higher in FW; these genes were from the Secreted Protein Acidic and Rich in Cysteine (SPARC) family, SPARC and testican–2 (or SPOCK2). These proteins modulate cellular interaction with the extracellular matrix by regulating ECM turnover, growth factor signaling and receptor activity, and extracellular protease activity [54]. Most of these genes were up-regulated in *Salmo salar* smolts compared to parr [55]. The smolt is one of last stages of smoltification (when salmon are ready to live in sea water), in contrast to the parr that lives only in fresh water, and our results appear opposed to this study. However, in Seear et al. (2010), transcriptome analysis was performed on whole gill tissue, whereas we worked with isolated ionocytes. Furthermore, in this previous study, smolts were only pre-acclimated to sea water because they were maintained in fresh water. The measurement of collagen and SPARC mRNA in smolts transferred to SW will be necessary to determine the importance of these genes in FW and SW. In our microarray results, the higher expression of two protease inhibitors, serine protease inhibitor Kazal-type 2 (SPINK2) and Fetuin-B (FETUB), secreted in the extracellular portion of FW ionocytes, strengthens the importance of this extracellular matrix for these salt-absorbing cells.

In the cytoskeleton, the transcriptional expression of a protein implicated in cytoskeleton F-actin organization, bridging integrator 3 (BIN3) [56], was significant in FW vs SW ionocytes. Two actin-associated proteins were also up-regulated in FW ionocytes, transgelin (TAGLN) and coronin–6 (CORO6). Down regulation of transgelin has been shown to have an oncogenic effect and to modify extracellular remodeling and invasion [57]. Furthermore, coronin–6 modulates anchoring between the receptor (the acetylcholine receptor, in particular) and the actin cytoskeleton [58]. Several genes related to microtubules, such as tubulin alpha-1D chain (TUBA1D), and implicated in intracellular trafficking were also up-regulated in FW ionocytes: cytoplasmic dynein 1 intermediate chain 2 (DYNC1L2) [59], sortin nexin (SNX3), [50], vesicle-associated membrane protein 5 (VAMP5) [60] and spastin (SPAST) [61]. All genes suggest an important role of the cytoskeleton in FW ionocyte functions.

### Cell adhesion and motility

In relation to cell structure and morphogenesis, other genes implicated in cell adhesion and motility were up-regulated in freshwater ionocytes, including vascular cell adhesion protein 1 VCAM1), liprin-beta–1 (PPFIBP1), CD9 antigen (CD9) [62–64], as well as carcinoembryonic antigen-related cell adhesion molecule 5 (CEACAM5) [65]. Unlike in SW, where most of the gill ionocytes were localized on filaments, the ionocytes in FW rainbow trout were also localized in all parts of the lamellae [66]. Gill epithelial proliferation occurred mainly at the base of lamellae with minor proliferation at the base of the filament [67]. To be operational, ionocytes have to migrate from the progenitor compartment. This distance is more important to access the lamellae, which may explain the higher transcript expression for adhesion and motility in FW ionocytes.

Cell-cell adhesion also includes proteins that form tight junctions in the epithelium. Among these tight junction proteins, several claudins have been characterized [68]. In our study, higher RNA expression of claudin–4 (CLDN4) in FW ionocytes was in accordance with other studies showing a significant elevation of claudin 4 protein in the gills of tilapia, killifish, and southern flounder acclimated to FW versus SW [18,69,70]. Another protein from the apical intercellular junction in epithelial cells [71] was up-regulated in FW ionocytes: peripheral myelin protein 22 (PMP22). As another junction protein, expression of human PMP22 in cultured kidney cells (MDCK) elevated the transepithelial resistance but at the same time increased permeability of nonionic molecules [72]. Further experiments will be necessary to understand the role of PMP22 in gill epithelium.

### Energetic metabolism

Several enzymes implicated in mitochondrial ATP synthesis presented higher transcriptional expression in FW ionocytes, suggesting higher energy demand than the SW ionocytes. Among the genes implicated were (i) two enzymes in tricarboxylic acid cycle, isocitrate dehydrogenase (IDH2) and aconitate hydratase, and (ii) several proteins of the electron transfer chain, enzymes of complex I (NADH dehydrogenase [ubiquinone] flavoprotein 1 and 2 (NDUFV1 and NDUFV 2)) and complex III (Cytochrome b-c1 complex subunit (UQCRH)). Higher expression of glycogen phosphorylase (the cytoplasmic enzyme implicated in glycogen catabolism and consequent glucose increase) could link this to ATP production. A component and regulator of mitochondrial ATP synthase was also up-regulated, diabetes-associated protein in insulin-sensitive tissues (DAPIT also known as USMG5) [73]. This demand in ATP synthesis can be explained by higher ion-ATPase activity in fresh water. In FW and SW, Na/K-ATPase pumps are present in the basolateral plasma membrane of ionocytes. However, in FW rainbow trout, another ATPase pump has been shown to play a role in Na^+^ uptake, the H-ATPase pump [74]. The changes in carbohydrate metabolism of gills during the gradual adaptation of rainbow trout to sea water was measured by a previous study [75] that suggested a higher ATP demand to adapt to salt water. However, this study was performed on whole gill tissue during adaptation. The ionocytes represent less than 10% of the whole gill, thus their metabolic activity is most likely hidden by the global activity of whole gill tissue.

An increase of electron transport chain (ETC), in the mitochondria, for higher energy production can result in the formation of more reactive oxygen species (ROS) that contribute to oxidative stress [76]. Two mitochondrial enzymes identified as protective proteins during oxidative stress were expressed more in FW ionocytes, aldehyde dehydrogenase–2 (ALDH2) and NAD(P) transhydrogenase (NTT). ALDH2 detoxifies aldehydes produces by ROS [77], and NNT generates NADPH, a cofactor required for antioxidant-related enzyme activity, such as glutathione peroxidase and glutathione reductase [78]. Among the genes with higher expression in FW ionocytes, one other antioxidant protein was identified: hemoglobin beta (HBB) [79].

### Amino acid and protein metabolism

Several genes related to amino acid synthesis were also up-regulated in FW compared to SW ionocytes. These genes included 5-methyltetrahydropteroyltriglutamate-homocysteine methyltransferase (metE) and proline synthetase co-transcribed bacterial homolog protein (PROSC). Similar to data reported by Whitehead et al., ornithine decarboxylase 1 (ODC–1), implicated in polyamine synthesis, was up-regulated under hypoosmotic conditions.

Several genes implicated in protein synthesis were up-regulated in FW and SW ionocytes. In FW ionocytes, these genes included 2 genes involved in translation initiation, eukaryotic translation initiation factor 4E-1A (EIF4E1A) and ATP-binding cassette sub-family F member 1 (ABCF1), and for SW, another translation initiation factor and a ribosome protein, eukaryotic translation initiation factor 3 subunit B (EIF3B) and 40S ribosomal protein S7 (RPS7), respectively.

Other genes implicated in protein regulation were only enhanced in FW ionocytes; thus, several genes of the ubiquitin proteasome system (UPS) were up-regulated in FW versus SW ionocytes. Protein modified by ubiquitination, which is performed by activating (E1), conjugating (E2) and ligating (E3) enzymes, can be degraded or implicated in other functions, such as DNA repair, trafficking and endosomal sorting. The fate of ubiquitylated protein depends on the number of conjugated ubiquitin chains (mono or polyubiquitin chains) and on the amino acid residues of ubiquitin involved [80,81]. For example, a protein conjugated to a polyubiquitin chain by ubiquitin lysine 48 (but possibly by all non-lysine 63 linkage) is targeted to the 26S proteasome to be degraded [80]. In our microarray results, one E3 ubiquitin-protein ligase mediating 'Lys–48'-linked polyubiquitination of target proteins (RING finger protein 152 (RNF152)) [82] was up-regulated in FW ionocytes. Furthermore, two non-ATPase regulatory subunits of the 26S proteasome were concomitantly more expressed (PSMD5 and PSMD 7). Ubiquilins are ubiquitin-like proteins that play a role in the regulation of proteasomal protein degradation [83], and ubiquilin 4 (UBQLN4) appears up-regulated in FW ionocytes. All of these genes suggest protein degradation after ubiquitin conjugation. A higher rate of proteolysis in FW ionocytes is also suggested by the up regulation of one serine-protease, transmembrane protease serine 9 (TMPRSS9) [84].

Another UPS protein was more expressed in FW ionocytes, it was a E2-conjugating enzyme, NEDD8-conjugating enzyme (UBE2F). This enzyme interacts with E3 ubiquitin ligase, RBX2 [85]. Several factors can modify this enzyme interaction, defective in cullin neddylation protein 1-like protein 1 (DCUN1D) [86] and COP9 signalosome [87]. Among the genes up-regulated in FW ionocytes, we observed DCN1-like protein 4 (DCUN1D4) and the COP9 signalosome complex subunit 5 (COPS5).

## Conclusion

This study on gill ionocyte from acclimated trout (freshwater and seawater) has allowed (i) to compare transcriptome of cells absorbing versus cell secreting salts and (ii) to develop a LCM technique on trout gill epithelium. This study is the first to provide information specific to the functions of ionocytes (and not whole gill tissue) related to FW versus SW acclimation. Higher expression of genes in FW cells suggests higher activity of ionocyte in fresh water compared to salt water. Interestingly, new ion transporters, such as CIC2 and SLC10A3, have been identified. Further characterization of these ion transporters is necessary. In perspective, the development of this LCM technique in fish gills can be adapted to (1) compare transcriptomic cell signatures of others cells (i.e., pavement cells and ionocyte subtypes with specific antibodies) but also to (2) monitor certain cell types during external challenges, such as the modification of salinity.

## Supporting Information

S1 Table List of oligonucleotides up (FC>3) or down (FC<3) regulated in freshwater ionocyte compared to seawater ionocyte. Ratios>3 identified transcripts that were upregulated in freshwater ionocyte and ratios<3 identified transcripts that were upregulated in seawater ionocytes.
(DOCX)
