# Supplementary material for: Transcriptomic Analysis of Trout Gill Ionocytes in Fresh Water and Sea Water Using Laser Capture Microdissection Combined with Microarray Analysis
Source: PLoS One. 2015 Oct 6;10(10):e0139938. doi: 10.1371/journal.pone.0139938 (PMC4595143; doi:10.1371/journal.pone.0139938)
Supplement: S1 Table — Ratios>3 identified transcripts that were upregulated in freshwater ionocyte and ratios<3 identified transcripts that were upregulated in seawater ionocytes. (DOCX) [file pone.0139938.s001.docx]

| Oligo name | Gene identification | Abbreviation | Mean Fold Change FW/SW | Function |
| --- | --- | --- | --- | --- |
| CUST_62_PI425536763 | [BBH] S4A4_RABIT (sp:Q9XSZ4) Electrogenic sodium bicarbonate cotransporter 1 OS=Oryctolagus cuniculus GN=SLC4A4 PE=1 SV=1 | slc4a4 | 16.8001 | ion transport |
| TC95085 | (sp:Q9YH26) Sodium/potassium-transporting ATPase subunit alpha-1 OS=Oreochromis mossambicus GN=atp1a1 PE=2 SV=2 | atp1a1 | 16.5711 | ion transport |
| CUST_195_PI425708691 | NBC | slc4a4 | 16.4003 | ion transport |
| CUST_11397_PI425536763 | (sp:P51789) Chloride channel protein 2 OS=Oryctolagus cuniculus GN=CLCN2 PE=2 SV=1 | CLCN2 | 15.4991 | ion transport |
| CUST_19538_PI425536763 | Oncorhynchus masou Na+K+-ATPase-alpha1a mRNA for sodium/potassium-transporting ATPase subunit alpha-1a, complete cds | atp1a1 | 14.0652 | ion transport |
| CUST_192_PI425708691 | Na/K ATPase -1a | atp1a1 | 13.1867 | ion transport |
| CUST_3840_PI425536763 | [BBH] S26A6_HUMAN (sp:Q9BXS9) Solute carrier family 26 member 6 OS=Homo sapiens GN=SLC26A6 PE=1 SV=1 | SLC26A6 | 10.9978 | ion transport |
| CUST_20925_PI425536763 | (sp:Q3T0G5) Proline synthetase co-transcribed bacterial homolog protein OS=Bos taurus GN=PROSC PE=2 SV=1 | PROSC | 9.9724 | amino acid synthesis |
| TC104822 | Oncorhynchus mykiss sodium bicarbonate cotransporter (nbc), mRNA | slc4a4 | 9.0926 | ion transport |
| TC107002 | (sp:P35615) Eukaryotic peptide chain release factor subunit 1 OS=Xenopus laevis GN=etf1 PE=2 SV=1 | etf1 | 8.7997 | RNA catabolic process |
| TC122131 | (sp:P18745) Oocyte zinc finger protein XlCOF22 OS=Xenopus laevis PE=3 SV=1 |  | 7.5596 |  |
| TC103721 | (sp:P11024) NAD(P) transhydrogenase, mitochondrial OS=Bos taurus GN=NNT PE=1 SV=3 | NNT | 7.5237 | response to oxygen level |
| TC107441 | (sp:Q8CI59) Metalloreductase STEAP3 OS=Mus musculus GN=Steap3 PE=1 SV=1 | Steap3 | 7.4003 | iron homeostasis |
| TC109122 |  |  | 7.3829 |  |
| TC123964 | (sp:Q9I9M5) Frizzled-1 OS=Xenopus laevis GN=fzd1 PE=2 SV=1 | fzd1 | 7.0169 | cell signaling |
| CUST_2285_PI425536763 | (sp:Q108U6) Hepatocyte growth factor receptor OS=Loxodonta africana GN=MET PE=3 SV=1 | MET | 6.9837 | cell signaling |
| TC100122 | Salmo salar Vesicle-associated membrane protein 5 (vamp5), mRNA | vamp5 | 6.9504 | cytoskeleton |
| TC131408 | (sp:Q9NRR5) Ubiquilin-4 OS=Homo sapiens GN=UBQLN4 PE=1 SV=2 | UBQLN4 | 6.8743 | protein metabolism |
| CUST_9631_PI425536763 | (sp:O55173) 3-phosphoinositide-dependent protein kinase 1 OS=Rattus norvegicus GN=Pdpk1 PE=2 SV=1 | Pdpk1 | 6.6855 | cell signaling |
| TC102531 | (sp:Q9QYF9) Protein NDRG3 OS=Mus musculus GN=Ndrg3 PE=1 SV=1 | Ndrg3 | 6.5923 | cell cycle-division |
| TC111830 | Danio rerio collagen, type I, alpha 1a (col1a1a), mRNA | col1a1 | 6.5196 | extracellular matrix |
| TC102822 | Salmo salar Growth factor receptor-bound protein 2 (grb2), mRNA | grb2 | 6.3886 | cell signaling |
| TC132314 | chloride channel 2 [Source:HGNC Symbol;Acc:2020] | CLCN2 | 6.3203 | Ion transport |
| TC110056 | (sp:Q54VI5) Uncharacterized protein DDB_G0280315 OS=Dictyostelium discoideum GN=DDB_G0280315 PE=4 SV=1 |  | 6.3044 |  |
| TC102531 | (sp:Q9QYF9) Protein NDRG3 OS=Mus musculus GN=Ndrg3 PE=1 SV=1 | Ndrg3 | 6.1167 | cell cycle-division |
| TC117357 | Oncorhynchus mykiss sodium bicarbonate cotransporter (nbc), mRNA | slc4a4 | 6.0818 | ion transport |
| TC109952 | [BBH] PLBL1_BOVIN (sp:Q9GL30) Putative phospholipase B-like 1 OS=Bos taurus GN=PLBD1 PE=2 SV=2 | PLBD1 | 6.065 | lipid metabolism |
| CUST_27733_PI425536763 | (sp:O88343) Electrogenic sodium bicarbonate cotransporter 1 OS=Mus musculus GN=Slc4a4 PE=1 SV=2 | Slc4a4 | 6.0083 | ion transport |
| TC108202 | (sp:Q04202) Transposable element Tcb2 transposase OS=Caenorhabditis briggsae PE=3 SV=1 |  | 5.9273 | DNA integration |
| TC106283 | (sp:Q9EQZ1) TSC22 domain family protein 3 OS=Rattus norvegicus GN=Tsc22d3 PE=2 SV=1 | Tsc22d3 | 5.9172 | regulation of transcription |
| TC105913 | (sp:P33198) Isocitrate dehydrogenase [NADP], mitochondrial (Fragment) OS=Sus scrofa GN=IDH2 PE=1 SV=1 | IDH2 | 5.7249 | energetic metabolism |
| CUST_6222_PI425536763 | (sp:Q8N8N0) RING finger protein 152 OS=Homo sapiens GN=RNF152 PE=2 SV=1 | RNF152 | 5.6363 | protein metabolism |
| TC130062 | sparc/osteonectin, cwcv and kazal-like domains proteoglycan (testican) 2 [Source:HGNC Symbol;Acc:13564] | Spock2 | 5.5763 | extracellular matrix |
| CUST_3976_PI425536763 | (sp:Q2HJ86) Tubulin alpha-1D chain OS=Bos taurus GN=TUBA1D PE=1 SV=1 | TUBA1D | 5.4805 | cytoskeleton |
| TC104952 | (sp:Q9JI08) Bridging integrator 3 OS=Mus musculus GN=Bin3 PE=2 SV=1 | Bin3 | 5.3772 | cytoskeleton |
| TC107792 | [BBH] CO1A1_CHICK (sp:P02457) Collagen alpha-1(I) chain OS=Gallus gallus GN=COL1A1 PE=1 SV=3 | COL1A1 | 5.2249 | extracellular matrix |
| TC121940 |  |  | 5.135 |  |
| CUST_17192_PI425536763 | [BBH] TIP_MACFA (sp:Q95KC8) T-cell immunomodulatory protein (Fragment) OS=Macaca fascicularis GN=ITFG1 PE=2 SV=2 | ITFG1 | 5.0065 | immune system |
| TC104200 | (sp:P06731) Carcinoembryonic antigen-related cell adhesion molecule 5 OS=Homo sapiens GN=CEACAM5 PE=1 SV=2 | CEACAM5 | 4.9458 | cell adhesion |
| CUST_21914_PI425536763 | (sp:O88801) Homer protein homolog 2 OS=Rattus norvegicus GN=Homer2 PE=1 SV=1 | Homer2 | 4.8779 | cell signaling |
| TC109201 | [BBH] GBRL2_RAT (sp:P60522) Gamma-aminobutyric acid receptor-associated protein-like 2 OS=Rattus norvegicus GN=Gabarapl2 PE=2 SV=1 | Gabarapl2 | 4.8682 | cell signaling |
| TC112280 | predicted protein [Nematostella vectensis] |  | 4.8375 |  |
| TC126016 |  |  | 4.8305 |  |
| TC101055 | Salmo salar Uridine 5-monophosphate synthase (pyr5), mRNA | pyr5 | 4.8295 | protein metabolism |
| TC106801 | (sp:Q9JHW0) Proteasome subunit beta type-7 OS=Rattus norvegicus GN=Psmb7 PE=1 SV=1 | Psmb7 | 4.8228 | protein metabolism |
| CUST_20059_PI425536763 | (sp:Q712U5) cAMP-regulated phosphoprotein 19 OS=Rattus norvegicus GN=Arpp19 PE=1 SV=3 | Arpp19 | 4.799 | cell cycle_division |
| TC111031 | Danio rerio collagen, type I, alpha 2 (col1a2), mRNA | col1a2 | 4.7493 | extracellular matrix |
| TC112264 | (sp:Q28260) Vascular cell adhesion protein 1 OS=Canis familiaris GN=VCAM1 PE=2 SV=1 | VCAM1 | 4.7187 | cell adhesion |
| TC115030 | (sp:Q8C8U0) Liprin-beta-1 OS=Mus musculus GN=Ppfibp1 PE=1 SV=3 | Ppfibp1 | 4.6989 | cell adhesion |
| CUST_15937_PI425536763 | (sp:Q5RHX6) DCN1-like protein 4 OS=Danio rerio GN=dcun1d4 PE=4 SV=2 | dcun1d4 | 4.6966 | protein metabolism |
| TC109387 | Salmo salar CD9 protein (LOC100136380), mRNA | CD9 | 4.3949 | cell adhesion |
| TC118868 | (sp:P27117) Ornithine decarboxylase OS=Bos taurus GN=ODC1 PE=2 SV=1 | ODC1 | 4.3932 | amino acid synthesis |
| CUST_11446_PI425536763 | (sp:P69526) Transmembrane protease serine 9 OS=Rattus norvegicus GN=Tmprss9 PE=3 SV=1 | Tmprss9 | 4.3887 | protein metabolism |
| TC107703 | (sp:Q8N335) Glycerol-3-phosphate dehydrogenase 1-like protein OS=Homo sapiens GN=GPD1L PE=1 SV=1 | GPD1L | 4.3721 | carbohydrate and lipid metabolism |
| TC102439 |  |  | 4.3675 |  |
| CUST_4971_PI425536763 | (sp:P50430) Arylsulfatase B OS=Rattus norvegicus GN=Arsb PE=2 SV=2 | Arsb | 4.3358 | glucopolysaccharide catabolism |
| TC102463 | [BBH] QCR6_HUMAN (sp:P07919) Cytochrome b-c1 complex subunit 6, mitochondrial OS=Homo sapiens GN=UQCRH PE=1 SV=2 | UQCRH | 4.1836 | energetic metabolism |
| TC102053 | (sp:P26632) Early growth response protein 1 OS=Danio rerio GN=egr1 PE=2 SV=2 | egr1 | 4.1825 | regulation of transcription |
| TC103455 | Oncorhynchus mykiss C4b-binding protein alpha chain (c4bp), mRNA | c4bp | 4.1814 | immune system |
| TC102053 | (sp:P26632) Early growth response protein 1 OS=Danio rerio GN=egr1 PE=2 SV=2 | egr1 | 4.1427 | regulation of transcription |
| CUST_9735_PI425536763 | (sp:P27120) Ornithine decarboxylase 1 OS=Xenopus laevis GN=odc1-A PE=2 SV=1 | odc1-a | 4.1311 | amino acid synthesis |
| TC110202 | (sp:Q6NY82) NEDD8-conjugating enzyme UBE2F OS=Danio rerio GN=ube2f PE=2 SV=1 | ube2f | 4.1285 | protein metabolism |
| CUST_25811_PI425536763 | [BBH] METE_PSEF5 (sp:Q4KE22) 5-methyltetrahydropteroyltriglutamate--homocysteine methyltransferase OS=Pseudomonas fluorescens (strain Pf-5 / ATCC BAA-477) GN=metE PE=3 SV=1 | metE | 4.1232 | amino acid synthesis |
| TC131589 | (sp:Q535K8) GON-4-like protein OS=Rattus norvegicus GN=Gon4l PE=2 SV=1 | Gon4l | 4.0278 | regulation of transcription |
| TC102439 |  |  | 3.9714 |  |
| CUST_3562_PI425536763 | [BBH] ELOV1_HUMAN (sp:Q9BW60) Elongation of very long chain fatty acids protein 1 OS=Homo sapiens GN=ELOVL1 PE=1 SV=1 | ELOVL1 | 3.9193 | lipid metabolism |
| CUST_5876_PI425536763 | (sp:P19234) NADH dehydrogenase [ubiquinone] flavoprotein 2, mitochondrial OS=Rattus norvegicus GN=Ndufv2 PE=1 SV=2 | Ndufv2 | 3.9003 | energetic metabolism |
| TC102615 | [BBH] SPRC_RAT (sp:P16975) SPARC OS=Rattus norvegicus GN=Sparc PE=1 SV=4 | Sparc | 3.8699 | extracellular matrix |
| TC121733 |  |  | 3.8699 |  |
| CUST_1676_PI425536763 | (sp:Q9DFS6) Eukaryotic translation initiation factor 4E-1A OS=Danio rerio GN=eif4e1a PE=1 SV=1 | eif4e1a | 3.8639 | protein metabolism |
| TC112418 |  |  | 3.8361 |  |
| TC118602 | [BBH] HBB1_ONCMY (sp:P02142) Hemoglobin subunit beta-1 OS=Oncorhynchus mykiss GN=hbb1 PE=1 SV=1 | hbb1 | 3.8081 | response to oxygen level |
| TC106966 | (sp:P02452) Collagen alpha-1(I) chain OS=Homo sapiens GN=COL1A1 PE=1 SV=4 | COL1A1 | 3.7464 | extracellular matrix |
| TC114099 | (sp:P01893) Putative HLA class I histocompatibility antigen, alpha chain H OS=Homo sapiens GN=HLA-H PE=5 SV=2 | HLA-H | 3.7313 | immune system |
| TC104128 | [BBH] MAX_DANRE (sp:P52161) Protein max OS=Danio rerio GN=max PE=2 SV=1 | max | 3.7048 | regulation of transcription |
| TC102033 | [BBH] SNRPA_PIG (sp:Q06AA4) U1 small nuclear ribonucleoprotein A OS=Sus scrofa GN=SNRPA PE=2 SV=1 | SNRPA | 3.7023 | mRNA splicing |
| TC108897 | (sp:Q5RF00) Aldehyde dehydrogenase, mitochondrial OS=Pongo abelii GN=ALDH2 PE=2 SV=1 | ALDH2 | 3.702 | response to oxygen level |
| TC128891 | (sp:Q13247) Splicing factor, arginine/serine-rich 6 OS=Homo sapiens GN=SFRS6 PE=1 SV=2 | SFRS6 | 3.662 | mRNA splicing |
| TC116740 | [BBH] BI1_PAROL (sp:Q9IA79) Probable Bax inhibitor 1 OS=Paralichthys olivaceus GN=tmbim6 PE=2 SV=1 | tmbim6 | 3.6459 | cell cycle_anti-apoptosis |
| CUST_425_PI425536763 | (sp:P02751) Fibronectin OS=Homo sapiens GN=FN1 PE=1 SV=3 | FN1 | 3.6266 | extracellular matrix |
| CUST_13955_PI425536763 | (sp:Q16401) 26S proteasome non-ATPase regulatory subunit 5 OS=Homo sapiens GN=PSMD5 PE=1 SV=3 | PSMD5 | 3.6253 | protein metabolism |
| CUST_5542_PI425536763 | (sp:O93484) Collagen alpha-2(I) chain OS=Oncorhynchus mykiss GN=col1a2 PE=2 SV=2 | col1a2 | 3.614 | extracellular matrix |
| TC120460 | (sp:Q9CSH3) Exosome complex exonuclease RRP44 OS=Mus musculus GN=Dis3 PE=2 SV=4 | Dis3 | 3.5714 | RNA catabolic process |
| CUST_6915_PI425536763 | (sp:Q6MG08) ATP-binding cassette sub-family F member 1 OS=Rattus norvegicus GN=Abcf1 PE=1 SV=1 | Abcf1 | 3.5549 | protein metabolism |
| TC128878 |  |  | 3.5467 |  |
| TC132055 | Salmo salar RMD5 homolog B (rmd5b), mRNA | rmd5b | 3.5335 |  |
| TC105522 | Salmo salar Zinc transporter SLC39A11 (s39ab), mRNA | s39ab | 3.4564 | ion transport |
| TC108745 | Danio rerio far upstream element (FUSE) binding protein 1 (fubp1), mRNA | fubp1 | 3.4236 | regulation of transcription |
| TC108656 | (sp:P02457) Collagen alpha-1(I) chain OS=Gallus gallus GN=COL1A1 PE=1 SV=3 | COL1A1 | 3.4099 | extracellular matrix |
| TC111834 | [BBH] GNAI2_CANFA (sp:P38400) Guanine nucleotide-binding protein G(i) subunit alpha-2 OS=Canis familiaris GN=GNAI2 PE=2 SV=2 | GNAI2 | 3.4087 | cell cycle_division |
| CUST_24098_PI425536763 | (sp:Q9JJW3) Up-regulated during skeletal muscle growth protein 5 OS=Rattus norvegicus GN=Usmg5 PE=2 SV=1 | Usmg5 | 3.4046 | energetic metabolism |
| TC112702 | (sp:P40240) CD9 antigen OS=Mus musculus GN=Cd9 PE=1 SV=2 CD fait parti de la tetraspanin family | CD9 | 3.388 | cell adhesion |
| CUST_4021_PI425536763 | (sp:Q9WU63) Heme-binding protein 2 OS=Mus musculus GN=Hebp2 PE=2 SV=1 | Hebp2 | 3.385 | cell cycle_pro-apoptosis |
| TC107775 |  |  | 3.3821 |  |
| CUST_9639_PI425536763 | (sp:Q7ZY35) Protein LLP homolog OS=Xenopus laevis GN=llph PE=2 SV=1 | llph | 3.3783 | regulation of transcription |
| TC118856 | [BBH] CSN5_DANRE (sp:Q6PC30) COP9 signalosome complex subunit 5 OS=Danio rerio GN=cops5 PE=2 SV=1 | cops5 | 3.3618 | protein metabolism |
| TC131169 | Drosophila erecta GG19652 (DereGG19652), mRNA |  | 3.3566 |  |
| TC97074 | (sp:Q5U211) Sorting nexin-3 OS=Rattus norvegicus GN=Snx3 PE=1 SV=1 | Snx3 | 3.3522 | cytoskeleton |
| TC130310 |  |  | 3.3439 |  |
| TC107846 |  |  | 3.325 |  |
| TC110438 | (sp:P20155) Serine protease inhibitor Kazal-type 2 OS=Homo sapiens GN=SPINK2 PE=1 SV=2 | SPINK2 | 3.324 | protease inhibition |
| CUST_20828_PI425536763 | (sp:B2RYN7) Spastin OS=Rattus norvegicus GN=Spast PE=2 SV=1 | Spast | 3.3048 | cytoskeleton |
| TC112402 | (sp:Q9QX79) Fetuin-B OS=Rattus norvegicus GN=Fetub PE=2 SV=1 | Fetub | 3.3032 | protease inhibition |
| CUST_16130_PI425536763 | (sp:O19005) Claudin-4 OS=Cercopithecus aethiops GN=CLDN4 PE=2 SV=1 | CLDN4 | 3.2958 | tight junction |
| TC112692 | [BBH] DC1I2_PONAB (sp:Q5NVM2) Cytoplasmic dynein 1 intermediate chain 2 OS=Pongo abelii GN=DYNC1I2 PE=2 SV=1 | DYNC1I2 | 3.2866 | cytoskeleton |
| TC110030 | (sp:Q9I9C3) Sodium/potassium-transporting ATPase subunit beta-233 OS=Anguilla anguilla GN=atnb233 PE=1 SV=1 | atnb233 | 3.272 | ion transport |
| TC110607 | (sp:P56517) Histone deacetylase 1 OS=Gallus gallus GN=HDAC1 PE=2 SV=1 | HDAC1 | 3.2427 | regulation of transcription |
| CUST_11707_PI425536763 | [BBH] PGAP3_DANRE (sp:A8WFS8) Post-GPI attachment to proteins factor 3 OS=Danio rerio GN=pgap3 PE=2 SV=1 | pgap3 | 3.2371 | GPI anchor metabolic process |
| TC120553 | Salmo salar Gametogenetin-binding protein 2 (ggnb2), mRNA | ggnb2 | 3.2253 | cell cycle_division |
| TC102676 |  |  | 3.2167 |  |
| TC126072 | [BBH] NDUV1_PONPY (sp:Q0MQI4) NADH dehydrogenase [ubiquinone] flavoprotein 1, mitochondrial OS=Pongo pygmaeus GN=NDUFV1 PE=2 SV=1 | NDUFV1 | 3.2003 | energetic metabolism |
| TC106217 | (sp:Q8K214) Polycomb protein SCMH1 OS=Mus musculus GN=Scmh1 PE=1 SV=1 | Scmh1 | 3.182 | regulation of transcription |
| TC105676 | (sp:P61805) Dolichyl-diphosphooligosaccharide--protein glycosyltransferase subunit DAD1 OS=Rattus norvegicus GN=Dad1 PE=2 SV=3 (defender against cell death) | Dad1 | 3.1502 | cell cycle_anti-apoptosis |
| TC106909 | (sp:Q6QEF8) Coronin-6 OS=Homo sapiens GN=CORO6 PE=1 SV=2 | CORO6 | 3.0897 | cytoskeleton |
| TC111682 | (sp:P09131) P3 protein OS=Homo sapiens GN=SLC10A3 PE=2 SV=1 | SLC10A3 | 3.0783 | ion transport |
| CUST_8539_PI425536763 | (sp:P49609) Aconitate hydratase, mitochondrial OS=Gracilaria verrucosa PE=3 SV=1 |  | 3.0739 | energetic metabolism |
| CUST_26916_PI425536763 | Transgelin [Salmo salar] | TAGLN | 3.0707 | cytoskeleton |
| TC108702 | collagen, type I, alpha 1b [Source:ZFIN;Acc:ZDB-GENE-030131-4400] | col1a1b | 3.0705 | extracellular matrix |
| TC102540 | (sp:Q9R1R0) LIM/homeobox protein Lhx6 OS=Mus musculus GN=Lhx6 PE=1 SV=1 | Lhx6 | 3.0677 | regulation of transcription |
| TC105560 | (sp:Q9TQZ3) Peripheral myelin protein 22 OS=Bos taurus GN=PMP22 PE=1 SV=2 | PMP22 | 3.0677 | tight junction |
| TC111094 |  |  | 3.0637 |  |
| TC109767 |  |  | 3.0589 |  |
| TC108586 | (sp:Q76LC6) RNA-binding protein 24 OS=Danio rerio GN=rbm24 PE=2 SV=1 | rbm24 | 3.0546 | cell cycle_differentiattion |
| TC128210 | (sp:O18751) Glycogen phosphorylase, muscle form OS=Ovis aries GN=PYGM PE=2 SV=3 | PYGM | 3.0523 | glycogen metabolism |
| CUST_7524_PI425536763 | [BBH] WNT11_COTJA (sp:P51891) Protein Wnt-11 OS=Coturnix coturnix japonica GN=WNT11 PE=2 SV=1 | WNT11 | 3.0353 | cell signaling |
| TC105917 | (sp:Q5R8G5) Major facilitator superfamily domain-containing protein 1 OS=Pongo abelii GN=MFSD1 PE=2 SV=1 | MFSD1 | 3.0241 | transporter |
| TC100541 | (sp:P23206) Collagen alpha-1(X) chain OS=Bos taurus GN=COL10A1 PE=2 SV=1 | COL10A1 | 3.0191 | extracellular matrix |
| TC103265 |  |  | 3.019 |  |
| CUST_5573_PI425536763 | (sp:P35615) Eukaryotic peptide chain release factor subunit 1 OS=Xenopus laevis GN=etf1 PE=2 SV=1 | etf1 | 3.0171 | RNA catabolic process |
| CUST_13686_PI425536763 | [BBH] HLF_HUMAN (sp:Q16534) Hepatic leukemia factor OS=Homo sapiens GN=HLF PE=2 SV=1 | HLF | 3.0015 | regulation of transcription |
| TC124445 | (sp:Q810T5) Histone acetyltransferase MYST2 OS=Rattus norvegicus GN=Myst2 PE=2 SV=1 | myst2 | -3.011 | regulation of transcription |
| TC100769 | (sp:Q6DHN0) Transmembrane protein 53 OS=Danio rerio GN=tmem53 PE=2 SV=1 | tmem53 | -3.0822 |  |
| TC101514 | [BBH] TMX1_BOVIN (sp:Q0Z7W6) Thioredoxin-related transmembrane protein 1 OS=Bos taurus GN=TMX1 PE=2 SV=1 | TMX1 | -3.2051 | regulation of transcription |
| CUST_5359_PI425536763 | (sp:P01850) T-cell receptor beta chain C region OS=Homo sapiens GN=TRBC1 PE=1 SV=2 | TRBC1 | -3.2615 | immune system |
| CUST_16209_PI425536763 | (sp:Q28G71) Forkhead box protein N3 OS=Xenopus tropicalis GN=foxn3 PE=2 SV=1 | foxn3 | -3.4427 | regulation of transcription |
| CUST_17997_PI425536763 | (sp:A6NKQ3) Uncharacterized protein ENSP00000361571 OS=Homo sapiens PE=4 SV=2 | WDR65 | -3.4907 | cytoskeleton |
| TC105248 | (sp:P13913) Arylamine N-acetyltransferase, pineal gland isozyme NAT-10 OS=Gallus gallus PE=2 SV=1 | NAT-10 | -3.5153 | xenobiotic detoxification |
| TC109977 |  |  | -3.6067 |  |
| TC129761 | (sp:Q90YR7) 40S ribosomal protein S7 OS=Ictalurus punctatus GN=rps7 PE=2 SV=1 | rps7 | -3.6171 | protein metabolism |
| CUST_4193_PI425536763 | [BBH] SELM_DANRE (sp:Q802G7) Selenoprotein M OS=Danio rerio GN=sepm PE=2 SV=2 | sepm | -3.779 | response to oxygen level |
| CUST_25800_PI425536763 | (sp:Q569Z1) Eukaryotic translation initiation factor 3 subunit B OS=Xenopus laevis GN=eif3b PE=2 SV=1 | eif3b | -5.1939 | protein metabolism |

Table S1. List of oligonucleotides up (FC>3) or down (FC<3) regulated in freshwater ionocyte compared to seawater ionocyte. Ratios>3 identified transcripts that were upregulated in freshwater ionocyte and ratios<3 identified transcripts that were upregulated in seawater ionocytes.

Oligos TC102053, TC102439, TC102531 were represented twice on the chip.
